# MultiTEP-Based Vaccines Targeting SARS-CoV-2 Spike Protein IgG Epitopes Elicit Robust Binding Antibody Titers with Limited Virus-Neutralizing Activity

**DOI:** 10.3390/pathogens13060520

**Published:** 2024-06-20

**Authors:** Tatevik Antonyan, Garri Chilingaryan, Karen Zagorski, Manush Ghazaryan, Armine Hovakimyan, Hayk Davtyan, Irina Petrushina, Olga King, Roman Kniazev, Nikolai Petrovsky, Anahit Ghochikyan

**Affiliations:** 1 Department of Molecular Immunology, The Institute for Molecular Medicine, Huntington Beach, CA 92647, USA; antonyan.tatevik2@gmail.com (T.A.);; 2 Bill Gross Stem Cell Research Center, University of California, Irvine, CA 92697, USA; 3 Institute for Memory Impairments and Neurological Disorders, University of California, Irvine, CA 92697, USA; 4 Vaxine Pty Ltd., 11 Walkley Avenue, Warradale, SA 5046, Australia

**Keywords:** COVID-19, Spike protein, epitope vaccines, MultiTEP platform, immunogenicity, neutralization

## Abstract

Within the last two decades, SARS-CoV-2 was the third zoonotic severe acute respiratory betacoronavirus (sarbecovirus) to infect humans, following SARS and MERS. The disruptions caused by the pandemic underscore the need for a universal vaccine against respiratory betacoronaviruses. Our group previously developed the universal platform for vaccine development, MultiTEP, which has been utilized in this study to generate a range of SARS-CoV-2 epitope vaccine candidates. We prepared and characterized 18 vaccines incorporating small peptide fragments from SARS-CoV-2 Spike protein fused with the MultiTEP sequence using overlapping PCR. Wild-type mice were immunized intramuscularly with the immunogen formulated in AdvaxCpG adjuvant. Serum antibodies were detected by ELISA, surrogate neutralization, and pseudovirus neutralization assays. Finally, the most promising vaccine candidate was administered to three non-human primates. All vaccines generated high titers of spike-binding IgG antibodies. However, only three vaccines generated antibodies that blocked RBD binding to the ACE2 receptor in a surrogate virus neutralization assay. However, none of the vaccines induced antibodies able to neutralize pseudotype viruses, including after the administration of the lead vaccine to NHPs. MultiTEP-based COVID-19 vaccines elicited robust, IgG-binding responses against the Spike protein in mice and non-human primates, but these antibodies were not neutralizing, underscoring the need to refine this approach further.

## 1. Introduction

By January 2023, over 775 million people had been affected, and around 7.05 million had died, from severe acute respiratory syndrome coronavirus 2 (SARS-CoV-2) infection. [1]

COVID-19 is the fifth documented pandemic since the 1918 flu pandemic [2]. SARS-CoV-2 is closely related to severe acute respiratory syndrome coronavirus (SARS, first reported in 2003) and Middle Eastern respiratory syndrome (MERS, first detected in 2012) [2,3,4]. Importantly, all three pathogens belong to the Betacoronavirus genus and Sarbecovirus subgenus. Inside the host, the SARS-CoV-2 virus undergoes rapid replication and can cause severe pneumonia and, in some cases, cytokine storm syndrome [5,6]. COVID-19 may cause acute respiratory distress syndrome (ARDS), interstitial lung disease, sepsis, heart failure, stroke, encephalopathy, and several persistent physical and mental health complications. Th17 cells play an important role in the induced autoimmune reaction and “cytokine storm syndrome” by activating the cytokine cascade, inhibiting Th1 differentiation, and suppressing Treg cells [5]. Immunocompromised individuals, including elderly people, showed especially high morbidity and mortality [7,8,9,10,11,12,13].

**Figure 1:**
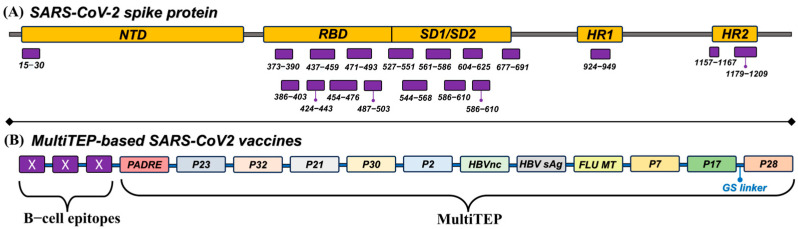
(**A**) Localization of selected IgG epitopes in SARS-CoV-2 Spike protein. NTD, N-terminal domain; RBD, receptor binding domain; SD1, subdomain 1; SD2, subdomain 2; HR1, heptad repeat 1 core peptide; HR2, heptad repeat 2 core peptide. (**B**) Schematic representation of the design of proposed MultiTEP-based vaccines. MultiTEP length is 228 a.a. and epitopes are around 20 a.a.

Current COVID-19 vaccines can mitigate severe disease, but are poorly effective against infection and transmission. Among them are inactivated virus-based vaccines, live attenuated vaccines, protein subunit vaccines, DNA vaccines, RNA vaccines, virus-like particles (VLP), non-replicating viral vector vaccines, and replicating viral vector vaccines [14]. Each of these technologies comes with unique advantages and disadvantages. The current mRNA and adenovirus-based vaccines have some concerns, including myocarditis, Guillain–Barré syndrome, and blood clotting disorders [15,16,17,18]. An epitope vaccine can target the immune response to specific neutralizing epitopes and can avoid immune-mediated disease enhancement [19,20,21,22,23]. Large numbers of peptide vaccine candidates have been described for HIV, influenza, COVID-19, and cancers, especially melanoma, breast, prostate, and lung cancer [24]. This study, thereby, sought to create an epitope vaccine targeting SARS-CoV-2. Peptides under 25 amino acids in length have low immunogenicity, but this can be overcome by conjugating them to a larger carrier protein. Our team has pioneered a highly immunogenic protein platform called MultiTEP that incorporates 12 foreign promiscuous CD4^+^ Th epitopes in a linear string (as illustrated in Figure 1B).

Given the role of the viral spike (S) glycoprotein in receptor binding and cellular entry, we designed 18 genetic constructs encoding diverse B-cell epitopes from S protein (Supplementary Figure S2) [25,26,27,28,29] fused with the MultiTEP sequence. All vaccines were formulated with AdvaxCpG adjuvant and evaluated in mice. Advax-CpG55.2 (VO_0005324 [30]) is a combination adjuvant comprising delta inulin (VO_0005207 [31]) and CpG55.2, an artificial intelligence-designed human Toll-like receptor 9 (TLR-9) agonist developed through the National Institutes of Health’s Adjuvant Discovery program [32]. Advax-CpG enhances vaccine protection by boosting humoral and T cell responses [33,34,35,36] while having low reactogenicity, and was previously successfully used in SARS [37] and MERS [38] coronavirus vaccines and also in the SpikoGen^®^ COVID-19 vaccine, which was licensed in the Middle East in October 2021 [39].

The most promising MultiTEP S peptide candidate vaccine, chosen based on the spike-binding IgG and surrogate viral neutralization (sVNT) results, underwent further evaluation in non-human primates (NHPs). While this candidate triggered high levels of spike-binding IgG in both mice and monkeys, the antibodies lacked virus-neutralizing activity, as assessed by pVNT assays, indicating the necessity for further optimization.

## 2. Materials and Methods

### 2.1. Animals

Mice: Female 6–8-week-old C57BL/6 mice (H-2b haplotype) were obtained from Jackson Laboratory, Sacramento, CA, USA. All animals were housed in a temperature- and light-cycle-controlled facility, and their care was under the guidelines of the NIH and an approved IACUC protocol at UC Irvine, CA, USA. Animal experiments were performed according to the guidelines of the Animal Care and Use Committee of UCI, and approved by University Laboratory Animal Resources (ULAR).

Non-human primates. Three adult (two females, one male) genetically unselected cynomolgus monkeys (*Macaca fascicularis*), ranging in age from 19 to 20 years, were obtained and housed at the primate colony at Alpha Genesis, Inc. (Yemassee, SC, USA). All animal procedures were conducted in an AAALACi-accredited facility in compliance with the Animal Welfare Act and other federal statutes and regulations relating to animals and experiments involving animals, per the Institutional Animal Care and Use Committee (IACUC) at Alpha Genesis, Inc. (Yemassee, SC, USA).

### 2.2. Epitope Vaccines

Eighteen minigenes, each encoding a protein composed of 3 copies of 1 of 18 various SARS-CoV-2 Spike protein epitopes fused to the N-terminus of MultiTEP with a histidine tag, were cloned into the *E. coli* expression vector pET24a. DNA sequencing was performed to confirm that the generated plasmids contained the correct sequences. Recombinant proteins were purified from *E. coli* BL21 (DE3) cells transformed with plasmids. Cells were grown up to the optical density 0.7–0.8 at 600 nm in Luria Bertani medium containing 100 µg mL^−1^ of kanamycin. Gene expression was induced by adding isopropyl-β-D-1-thiogalactopyranoside at a final concentration of 1 mM, and incubating for 4 h at 28 °C. Cells harvested by centrifugation were re-suspended in the lysis buffer, and the integrity of those cells was disrupted using sonication. The antigens appeared as inclusion bodies and were separated from cell debris using the BugBuster protein extraction reagent, as recommended by the manufacturer (Novagen, New Canaan, CT, USA). The final protein pellet was dissolved in 8 M of urea buffer (pH 8) and purified using a Ni-NTA agarose column (Qiagen, Valencia, CA, USA). Positive fractions were combined, and the protein was refolded by dilution in a refolding solution (4M urea, 1M L-Arginine HCl, 0.5% sarkosyl, pH 10), followed by gradual dialysis against 1× PBS, 0.5% sarkosyl to enhance the solubility of proteins. Since, under alkaline conditions, the urea can react with primary amines of free N-termini and ε-amine groups of lysines to form carbamyl derivatives, potentially resulting in structural alterations to the protein [40], all sample manipulations were conducted at a temperature of 4 °C or placed on ice for a short duration. This approach facilitated effective solubilization while reducing the chances of protein modifications.

### 2.3. Vaccine Administration

**Figure 2:**
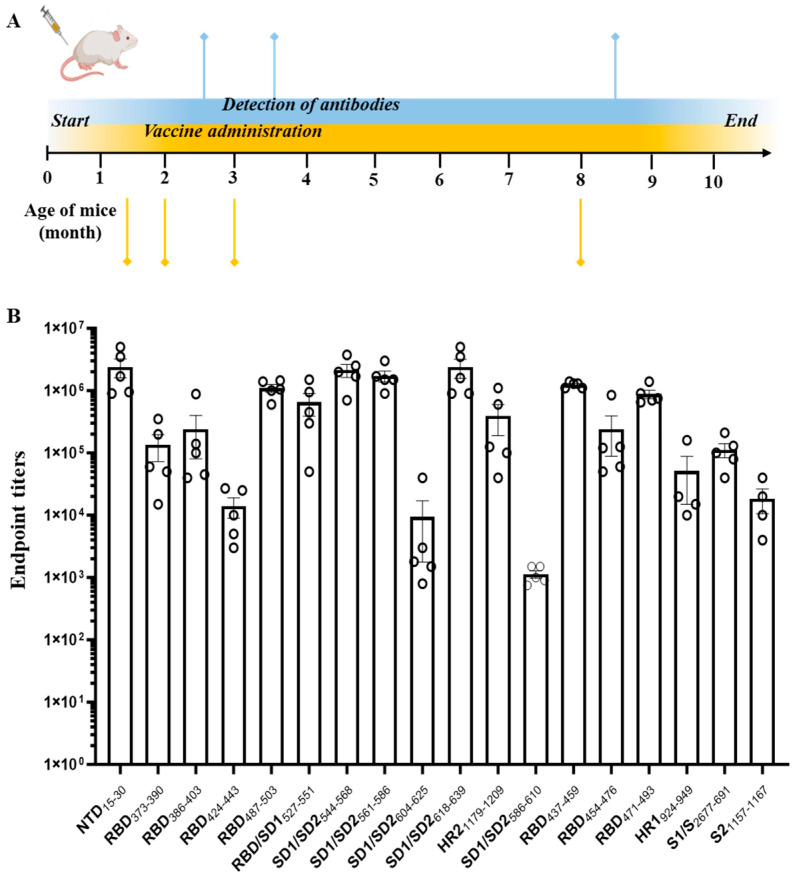
C57Bl/6 mice immunization schedule (**A**) and endpoint titers of 18 generated MultiTEP-based epitope vaccines (**B**). *n* = 5 mice per group. Bars show an average as mean ± SEM. ELISA assay was repeated independently three times with comparable results.

Mice experiment: 18 groups (*n* = 5 each group) of C57BL/6 mice (females, 2 months) were immunized intramuscularly with 18 different MultiTEP spike epitope vaccines (20 µg/mouse) formulated in AdvaxCpG adjuvant (1 mg/mouse) (Vaxine Pty Ltd., Adelaide, Australia). Each mouse received a total of four injections. Serum samples were collected at 3 distinct time points: 14 days subsequent to the 2nd immunization, 14 days following the 3rd immunization, and once more, 14 days after the 4th immunization. More detailed experimental protocols are provided in Figure 2.

NHP experiment: Three adult cynomolgus monkeys (*Macaca fascicularis*) were injected with 100 µg of MultiTEP-RBD471-493 vaccine formulated in AdvaxCpG adjuvant (10 mg Advax mixed with 100 µg CpG per dose) at weeks 0, 2, and 6. Monkeys were housed according to the accepted standards, and observed once daily for abnormal clinical signs or signs of illness or distress, including food intake, activity, appearance, and stool consistency. Body weights were measured before the first vaccine administration and at the time of subsequent administrations and blood draws.

### 2.4. Enzyme-Linked Immunosorbent Assay (Elisa)

Ninety-six-well high binding plates were separately coated with 1 μg/well (in 100 μL; Carbonate–Bicarbonate buffer, pH 9.6, o/n at 4 °C) of SARS-CoV-2 Spike protein S1 subunit (SinoBiological; #40591-V08B1) and the whole SARS-CoV-2 Spike monomer (SinoBiological; #40589-V08B1). The next day, the coated plates were washed three times and blocked with blocking buffer (3% dry, non-fat milk in TBST, 200 μL/well, o/n at 4 °C). Sera samples were diluted 3-fold serially, starting at 5000 or 25,000 (in 0.3% dry, non-fat milk in TBST, 100 μL/well), and then added to the plates and incubated o/n at 4 °C. The plates were washed three times with TBST. Then, HRP-conjugated goat anti-mouse IgG (Jackson ImmunoResearch Laboratories 1:2500 dilution) (cat#115-036-003) was added and incubated for 1 h at room temperature. The plates were washed three times with TBST before adding 3,3′,5,5′-tetramethylbenzidine (TMB) substrate solution (ThermoFisher, Cat# 34029). The reaction was stopped after 10 min by adding 2 N sulfuric acid. The OD at 450 nm was measured with a FilterMax F5 microplate reader. Endpoint titers of antibodies in mice sera were calculated as the reciprocals of the highest sera dilutions that provided an optical density reading thrice above the cutoff. The cutoff was determined as the titer of pre-immune sera at the same dilution.

### 2.5. Epitope Mapping of SARS-CoV-2-Specific Antibodies

Epitope mapping of anti-SARS-CoV-2 antibodies was performed by “alanine scanning” using competitive ELISA. Twenty-three peptides spanning the RBD471-493 (EI6GVEGFNCYFPLQ) sequence, but possessing one alanine substitution in each position, were synthesized.

Ninety-six-well plates (Thermofisher, Immulon 2HB, Cat#3455) were coated with SARS-CoV-2 Spike protein S1 subunit (SinoBiological; #40591-V08B1) at 1 μg/well (in 100 μL; Carbonate–Bicarbonate buffer, pH 9.6, o/n at 4 °C). The next day, the coated plates were blocked with blocking buffer (3% dry, non-fat milk in TBST, 200 μL/well). Serial dilutions of reference wild-type COVID-19 epitope or mutated test peptides (corresponding to 0.1 μM, 0.57 μM, 2.5 μM, 5 μM, and 12.5 μM final concentrations) were incubated with immune sera diluted 1:50,000 (corresponding to the linear region of the curve for binding to SARS-CoV-2 Spike protein S1 subunit) for 1.5 h at 37 °C. After incubation, 100 μL of antibody/peptide mixture was added into the wells. HRP-conjugated goat anti-mouse IgG (1:2500; Jackson ImmunoResearch Laboratories) was used as the secondary antibody.

The reaction was developed by adding 3,3′,5,5′ tetramethylbenzidine (TMB) substrate solution and stopped with 2 N H_2_SO_4_. The optical density (OD) was read at 450 nm using a FilterMax F5 microplate reader. The percent of the binding of sera blocked with wild-type or mutated peptides to SARS-CoV-2 Spike protein S1 subunit was calculated, relative to the binding of sera without competing peptides to SARS-CoV-2 Spike protein S1, as 100%. The half-maximal inhibitory concentration (IC50) for each peptide was calculated.

### 2.6. Surrogate Virus Neutralization Test (sVNT)

This blocking ELISA (GenScript, Cat# L00847-A) qualitatively detects anti-SARS-CoV-2 Abs, suppressing the interaction between the RBD of the viral spike glycoprotein (S) and the ACE2 protein on the surface of cells. After the preincubation of immune sera and control (all at a final dilution of 1:20, accounting for the initial sample dilution and the subsequent 1:2 dilution with RBD-HRP) with HRP–RBD, which allows Abs in the serum to bind to an HRP-conjugated RBD fragment (HRP–RBD), the mixture is added to a capture plate coated with human ACE2 protein. Any unbound HRP–RBD or HRP–RBD bound to non-neutralizing Abs is captured on the plate. Complexes of neutralizing Abs and HRP–RBD do not bind on the plate and are removed after three washing steps. Then, TMB is added as a substrate, allowing HRP to catalyze a color reaction. The color of the solution changes from blue to yellow after the addition of the stop reagent, and can be read by a microtiter plate reader at an OD of 450 nm (OD450). The absorbance of the sample is inversely correlated with the amount of SARS-CoV-2-neutralizing Abs. For final interpretation, the inhibition rates were determined using the following formula: Inhibition score (percentage) = (1 − (OD valuesample/OD valuenegative control) × 100%).

### 2.7. Pseudovirus Neutralization Assay

GenScript ProBio (Piscataway, NJ, USA) conducted the SARS-CoV-2 pseudovirus neutralization test. They designed a model simulating SARS-CoV-2 infection using lentivirus with substituted envelop glycoprotein with the S protein. Neutralizing antibodies block the S protein and receptor binding, preventing viral infection of ACE2-overexpressing HEK293T cells. The detection of target cell luminescence enabled the screening and verification of neutralizing antibody activity.

## 3. Results

### 3.1. Selection of Linear Epitopes and Preparation of the Immunogens

Eighteen spike epitopes were selected following a comprehensive literature review (Figure 1A). The chosen B-cell epitopes comprised in silico predicted sequences, sequences resembling known SARS-CoV-neutralizing epitopes, or those critical for the biological functionality of the Spike protein (Supplementary Table S1). Some sequences were split into fragments under 30 residues in length to minimize the risk of conformational neoepitope formation.

Every antigen featured three copies of the IgG epitopes, which were conjoined and then linked to the MultiTEP platform (Figure 1B). Additionally, a 6xHis-tag was positioned at the C-terminus of the construct for downstream purification. Multivalent antigen presentation can help stimulate the immune system by increasing the cross-linking of the B-cell receptors by the antigen [41]. Fusion proteins were expressed and purified from *E. coli* BL21 (DE3) cells and analyzed using 10% Bis-Tris gel electrophoresis (NuPAGE Novex Gel, Invitrogen^TM^ (Carlsbad, CA, USA). Protein bands were visualized by Coomassie dye (Supplementary Figure S1).

### 3.2. Immunogenicity of Recombinant Protein Epitope Vaccines in Mice

Eighteen groups of C57Bl/6 mice, five animals each, were immunized with vaccines formulated with AdvaxCpG adjuvant, according to the immunization schedule described in the Materials and Methods section and Figure 2A. All of the vaccines were well tolerated, with no adverse events noted. Sera samples were collected from the immunized mice for the downstream analysis. Binding antibodies specific to Spike protein were determined in collected sera by ELISA. As shown in Figure 2B and Supplementary Table S2, the majority of MultiTEP-based vaccines, with only a few exceptions, generated high IgG titers against the entire Spike protein.

**Figure 3:**
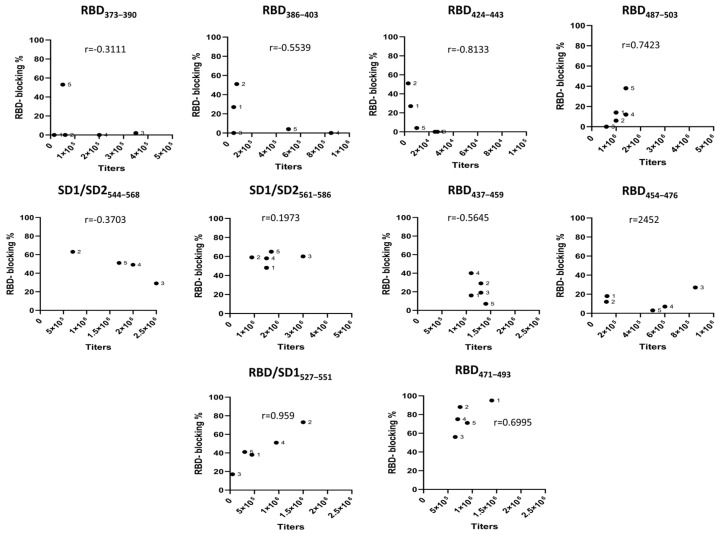
The analysis revealed that there was a correlation between antibody titers and blocking activity of immune sera in three experimental groups, RBD487-503, RBD/SD1527-551 and RBD471-493, with Pearson coefficients of r = 0.7423, r = 0.959, and r = 0.6995, respectively. For all mouse sera in the blocking activity assay, the final dilution was 1:20, which accounted for both the initial sample dilution and the subsequent 1:2 dilution with RBD-HRP.

To further assess the therapeutic potential of the generated vaccines, we used a surrogate neutralization assay based on the ability of antibodies to block the binding of ACE2 to RBD in an ELISA formal assay. The activity of each sample and the endpoint titer against the spike for each mouse within the group was analyzed by linear regression, with the results plotted for each epitope (Figure 3). The correlation between binding antibody titers and the neutralizing activity of sera was found to be weak. Among the tested groups, three exhibited neutralizing activity, with the peptide spanning residues 471–493 demonstrating the most potent effect. Notably, this peptide was recently identified as a functionally important disulfide-bridged loop [42]. Based on these findings, the vaccine containing this specific peptide was chosen for further evaluation and development.

### 3.3. Evaluation of Immunogenicity of RBD471-493 Vaccine in NHP

**Figure 4:**
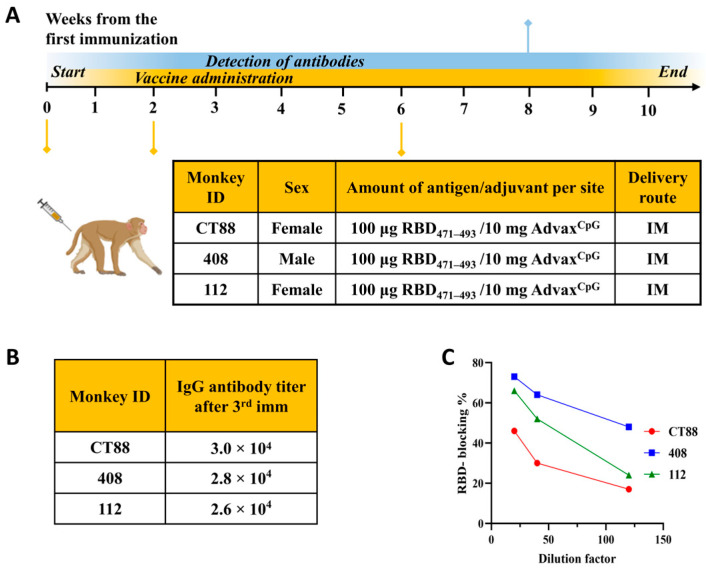
Three *Macaca fascicularis* (cynomolgus monkeys) received the RBD471-493 vaccine according to a designated immunization schedule (**A**). The table displays the titer of antibodies against the SARS-CoV-2 Spike protein in monkey sera following the third immunization (**B**). The antibodies produced after the third immunization inhibited the binding of RBD to hACE2. Sera dilutions accounted for the initial sample dilution and the subsequent 1:2 dilution with RBD-HRP (**C**). Assays were repeated twice.

To assess the immunogenicity of the vaccine, RBD471-493 was administered to three *Macaca fascicularis* (Cynomolgus monkeys) following the immunization schedule illustrated in Figure 4.

Following administration of the third RBD471-493 vaccine dose to the monkeys, a robust IgG binding response against Spike protein was observed in the sera of all test subjects, with endpoint titers ranging from 26,000 to 30,000 (Figure 4B). In the surrogate neutralization assay, monkey 112 demonstrated a 50% blocking capacity at 1:48, while monkey 408 exhibited a 50% blocking capacity at 1:105 (Figure 4C).

### 3.4. RBD471-493 Epitope Mapping Using an Alanine Scanning Competition ELISA

Antibodies generated in C57Bl6 mice against RBD471-493 were used to map the precise epitope through alanine scanning. The data showed that these antibodies specifically recognize an epitope encompassing amino acids 480–490 (CNGVEGFNCYF) within the non-mutated RBD471-493 peptide (Supplementary Figure S2A). Glycine 485 and Phenylalanine 486 were identified as the two most crucial amino acids playing a role in the binding of mouse anti-RBD471-493 antibodies to the epitope, with Leucine 492 also contributing to Spike protein binding (Supplementary Figure S2B).

Through alanine scanning, the same RBD471-493 B-cell epitope was mapped in the monkeys. The epitope in the monkeys comprised amino acids 480-488 (CNGVEGFNC), with minor variations observed among the experimental monkeys (Supplementary Figure S3). Interestingly, the epitope mapping of the monkey sera revealed that Cysteine 480 and Cysteine 488 played critical roles, suggesting a disulfide bond within the epitope. The recent crystal structure of the epitope confirms the existence of a disulfide bond between the cysteines and a loop-like structure [43].

### 3.5. Pseudovirus Neutralization Results

**Figure 5:**
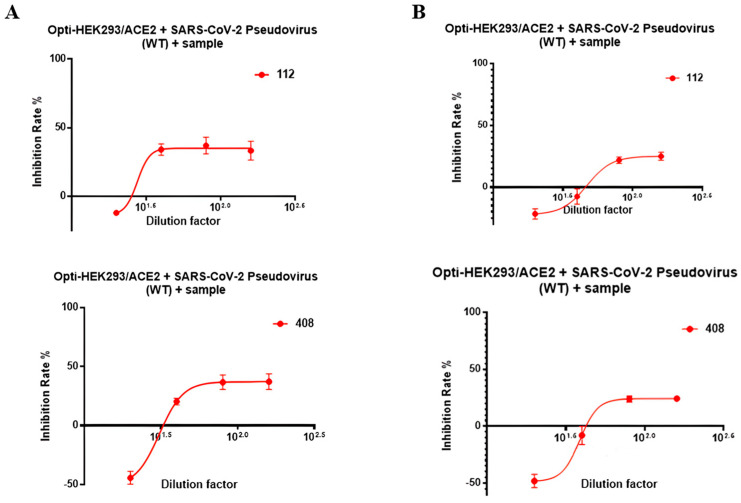
(**A**) The pseudovirus neutralization assay was performed on monkey sera generated against RBD471-493 without subjecting them to heat inactivation. (**B**) The pseudovirus neutralization assay was conducted on monkey sera generated against RBD471-493 after the samples were subjected to heat inactivation. The standard deviation (SD) in Figure 5 represents the variability between the four wells in each serum sample.

Many S epitopes are located outside the RBD, which limits the applicability of the RBD-based sVNT assay. This assay was developed using aldehyde–amine coupling of HRP to the RBD, potentially causing steric hindrance for certain epitopes due to lysine modification [44]. Notably, most epitopes within the RBD contain lysine residues. Consequently, we assessed the activity of sera generated by our vaccine candidates using a pseudovirus neutralization (pVNT) assay. We observed a trend of increased virus entry at lower serum dilutions, indicating that the antibodies might facilitate viral uptake into the cells, a phenomenon known as an antibody-dependent enhancement of infection. However, as the serum dilutions increased, a modest degree of viral inhibition was noted (Figure 5).

## 4. Discussion

Most MultiTEP-based vaccines formulated with AdvaxCpG adjuvant elicited robust binding antibody responses in the mice. The MultiTEP construct, RBD471-493, demonstrated the greatest ability to induce antibodies in mice that inhibited the binding of RBD to ACE2 by sVNT assay. RBD471-493 was then evaluated for immunogenicity in non-human primates. The immunization of monkeys with three doses of MultiTEP-RBD471-493 with AdvaxCpG adjuvant induced high IgG binding titers but low sVNT and pVNT titers. Indeed, there are published data by others suggesting antibody-dependent enhancement (ADE) at high serum concentrations, a phenomenon seen in the presence of predominantly non-neutralizing antibodies [45,46]. However, it is important to note that ADE has not been conclusively demonstrated in vivo for this particular context. For instance, studies have shown that while ADE can occur in vitro with antibodies isolated from patients infected with SARS-CoV-2 using artificially FcR/ACE2-expressing cells or Raji cells, there is no direct evidence that the ADE of disease is occurring in vivo [47]. Moreover, the clinical relevance of ADE is still under investigation, and more comprehensive studies are required to elucidate its impact in real-world scenarios [48]. Therefore, while the potential for ADE exists, its actual significance in vivo warrants further detailed research.

Pseudovirus neutralization was, at best, only partial, a phenomenon known as “incomplete neutralization” [49]. The conformation of epitopes within the RBD plays a pivotal role in virus attachment and infectivity through ACE2 binding [50]. Hence, the conformation of the peptide epitope may have changed from its native Spike structure when placed into the MultiTEP construct. Alternatively, more complete neutralization may require the inclusion of discrete S epitopes in the vaccine [49]. The effectiveness of our SARS-CoV-2 vaccines might be improved by developing antigens that present epitopes in a conformation better resembling their native S conformation. For instance, in the case of the antigen RBD471-493, it may be possible to design an antigen wherein this sequence assumes a native loop-like structure with a disulphite bond, mirroring its conformation in the native S protein. It is conceivable that the antibodies generated against our anti-Spike glycoprotein antigens displayed limited affinity for the native Spike protein due to a disparity in the conformation of the epitopes within our antigens compared to their conformation in the native Spike structure. A potential strategy to mitigate this concern involves a purposeful reduction in the length of epitopes chosen for our antigens. As elucidated in Supplementary Figure S3, when creating an antigen for the purpose of generating antibodies targeting the receptor binding domain (RBM) of the Spike protein, one viable option is to substitute the amino acid sequence encompassing 471–493 with the more concise epitope delineated by amino acids 480–490.

Furthermore, we have discerned the necessity for an antigen that amalgamates discrete epitopes from the Spike. This strategic approach aims to engineer an antigen capable of eliciting antibodies with the capacity to provide comprehensive coverage across all viral subgroups within a wild-type SARS-CoV-2 population.

It is noteworthy to emphasize that heat inactivation may exert a substantial influence on the dynamics of viral interactions with serum components, necessitating a deeper exploration. This is particularly pertinent when considering the integral role conformational epitopes occupy within the Spike protein’s structure. As such, further investigation into this phenomenon and optimization of the research methods are warranted.

In summary, we successfully synthesized a set of 18 different B-cell epitope protein vaccines targeting the S protein, utilizing the MultiTEP platform. The MultiTEP platform comprises 12 foreign promiscuous CD4+ T-helper (Th) epitopes recognized by various human MHC class II molecules. This was further boosted by the inclusion of the highly potent AdvaxCpG adjuvant. While highly immunogenic and able to induce high titers of binding antibodies, these epitope constructs failed to induce virus-neutralizing antibodies, indicating the importance of epitope selection and antigen conformation in designing an effective SARS-CoV-2 vaccine.

## Data Availability

All relevant data from this study are available from the authors.

## Supplementary Materials

The following supporting information can be downloaded at: https://www.mdpi.com/article/10.3390/pathogens13060520/s1, Figure S1: Analyses of purified proteins composed of indicated SARS-CoV-2 B-cell epitope fused with MultiTEP in SDS-PAGE in reducing conditions. Figure S2: The immune sera of mice immunized with RBD471-493 were pooled and subjected to epitope mapping using an alanine-scanning competition ELISA. Figure S3: Epitope mapping of immune sera from vaccinated *Macaca fascicularis* was performed by alanine-scanning competition ELISA. Table S1: The table displays information on the localization, peptide sequence, and selection method of various IgG epitopes derived from the Spike protein of SARS-CoV-2. Table S2: The IgG endpoint titer range within each group of C57Bl/6 mice 2 weeks after the third vaccination with MultiTEP-based SARS-CoV-2 vaccines.
