# Supplementary material for: MultiTEP-Based Vaccines Targeting SARS-CoV-2 Spike Protein IgG Epitopes Elicit Robust Binding Antibody Titers with Limited Virus-Neutralizing Activity
Source: Pathogens. 2024 Jun 20;13(6):520. doi: 10.3390/pathogens13060520 (PMC11206316; doi:10.3390/pathogens13060520)
Supplement: Supplementary file 1 [file pathogens-13-00520-s001.zip › pathogens-3028047-supplementary.pdf]

Supplementary Material

Supplementary Table S1. The table displays information on the localization, peptide sequence, and selection method of various IgG epitopes derived from the spike protein of SARS-CoV-2.

Vaccine ID	Region in S protein	Amino Acid Sequence	Epitope selection method	References
NTD_{15-30}	S1, NTD	15-CVNLTTRTQLPPAYTN-30	based on bioinformatic prediction	[25]
$RBD_{373-390}$	S1, RBD	373-SFSTFKCYGVSPTKLNDL-390	based on bioinformatic prediction	[25]
$RBD_{386-403}$	S1, RBD	386-KLNDLCFTNVYADSFVIR-403	based on homology with immunodominant region from SARS-CoV	[26]
$RBD_{424-443}$	S1, RBD/RBM	424-KLPDDFTGCVIAWNSNNLDS-443	based on homology with immunodominant region from SARS-CoV	[26]
$RBD_{487-503}$	S1, RBD/RBM	487-NCYFPLQSYGFQPTNGV-503	based on homology with immunodominant region from SARS-CoV	[28]
$RBD/SD1_{527-551}$	S1, RBD/SD1	527-PKKSTNLVKNKCVNFNFNGLTGTGV-551	based on bioinformatic prediction	[27]
$SD1/SD2_{544-568}$	S1, SD1/SD2	544-NGLTGTGVLTESNKKFLPFQQFGRD-568	based on bioinformatic prediction	[27]
$SD1/SD2_{561-586}$	S1, SD1/SD2	561-PFQQFGRDIADTTDAVRDPQTLEILD-586	based on bioinformatic prediction	[27]
$SD1/SD2_{604-625}$	S1, SD1/SD2	604-TSNQVAVLYQDVNCTEVPVAIH-625	based on homology with immunodominant region from SARS-CoV and bioinformatic prediction	[27, 28]
$SD1/SD2_{618-639}$	S1, SD1/SD2	618-TEVPVAIHADQLTPTWRVYSTG-639	based on homology with immunodominant region from SARS-CoV	[28]
$HR2_{1179-1209}$	S2, HR2	1179-EIDRLNEVAKNLNESLIDLQELGKYEQY-1209	based on homology with immunodominant region from SARS-CoV	[28]
$SD1/SD2_{586-610}$	S1, SD1/SD2	586-DITPCSFGGVSVITPGTNTSNQVAV-610	based on bioinformatic prediction	[27]
$RBD_{437-459}$	S1, RBD/RBM	437-NSNNLDSKVGGNYNYLYRLFRKS-459	based on the essential role of RBM in viral infection	
$RBD_{454-476}$	S1, RBD/RBM	454-RLFRKSNLKPFERDISTEIYQAG-476	based on the essential role of RBM in viral infection	
$RBD_{471-493}$	S1, RBD/RBM	471-EIYQAGSTPCNGVEGFNCYFPLQ-493	based on homology with immunodominant region from SARS-CoV	[28]
$HR1_{924-949}$	S2, HR1	924-ANQFNSAIGKIQDSLSSTASALGKLQ-949	based on the essential role of HR1 in viral infection	
$S1/S2_{677-691}$	Furin cleavage site	677-QTNSPRRARSVASQS-691	based on the importance of the cleavage step in viral infection	
$S2_{1157-1167}$	S2	1157-KNHTSPDVDLG-1167	based on the essential role of HR2 in viral infection	

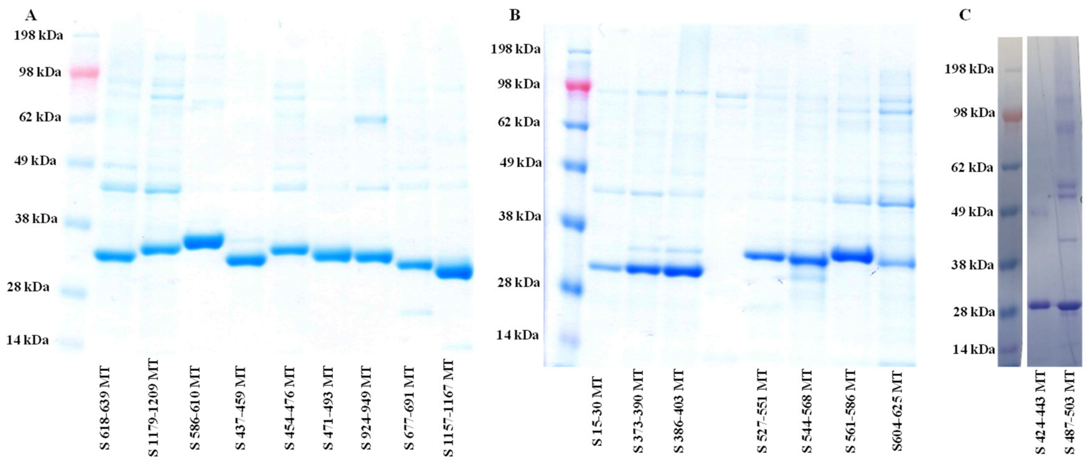

Supplementary Figure S1. Analyses of purified proteins composed of indicated SARS-CoV2 IgG epitopes fused with MultiTEP in SDS-PAGE in reducing conditions. (A, B, C) 3 µg of protein per well were loaded onto a 10% Bis-Tris gel.

Supplementary Table S2. The IgG endpoint titer range within each group of C57Bl/6 mice (n=5) 2 weeks after the third vaccination with MultiTEP-based SARS-CoV2 vaccines.

Vaccine ID	IgG antibody titer after 3rd imm	IgG antibody range per group
$NTD_{15\text{-}30}$	1.9E+06	9.0E+05 – 5.0E+06
$RBD_{373\text{-}390}$	7.9E+04	1.5E+04 – 3.5E+05
$RBD_{386\text{-}403}$	1.2E+05	4.0E+04 – 8.8E+05
$RBD_{424\text{-}443}$	1.0E+04	3.0E+03 – 2.7E+04
$RBD_{487\text{-}503}$	1.1E+06	6.0E+05 – 1.5E+06
$RBD/SD1_{527\text{-}551}$	4.0E+05	5.0E+04 – 1.5E+06
$SD1/SD2_{544\text{-}568}$	1.9E+06	7.0E+05 – 3.8E+06
$SD1/SD2_{561\text{-}586}$	1.6E+06	9.0E+05 – 3.0E+06
$SD1/SD2_{604\text{-}625}$	3.0E+03	1.5E+03 – 4.0E+04
$SD1/SD2_{618\text{-}639}$	1.9E+06	9.0E+05 – 5.0E+06
$HR2_{1179\text{-}1209}$	2.0E+05	6.0E+05 – 1.1E+06
$SD1/SD2_{586\text{-}610}$	1.1E+03	7.5E+02 – 1.5E+03
$RBD_{437\text{-}459}$	1.2E+06	1.1E+06 – 1.4E+06
$RBD_{454\text{-}476}$	1.3E+05	1.3E+05 – 8.5E+05
$RBD_{471\text{-}493}$	8.4E+05	6.5E+05 – 1.4E+06
$HR1_{924\text{-}949}$	1.9E+04	2.0E+04 – 1.6E+05
$S1/S2_{677\text{-}691}$	9.7E+04	4.0E+04 – 2.1E+05
$S2_{1157\text{-}1167}$	1.1E+04	4.0E+03 – 4.0E+04

	Peptide sequence	IC50 (µM)	Inhibition (%)
	EIYQAGSTPCNGVEGFNCYFPLQ	0.105	95%
E	AIYQAGSTPCNGVEGFNCYFPLQ	0.016	97%
I	EAYQAGSTPCNGVEGFNCYFPLQ	0.08	96%
Y	EIAQAGSTPCNGVEGFNCYFPLQ	0.035	97%
Q	EIYAAGSTPCNGVEGFNCYFPLQ	0.005	97%
A	EIYQSGSTPCNGVEGFNCYFPLQ	0.008	97%
G	EIYQAASTPCNGVEGFNCYFPLQ	0.026	97%
S	EIYQAGATPCNGVEGFNCYFPLQ	0.026	97%
T	EIYQAGSAPCNGVEGFNCYFPLQ	0.02	97%
P	EIYQAGSTACNGVEGFNCYFPLQ	0.192	97%
C	EIYQAGSTPANGVEGFNCYFPLQ	3.942	70%
N	EIYQAGSTPCAGVEGFNCYFPLQ	0.937	67%
G	EIYQAGSTPCNAVEGFNCYFPLQ	1.039	85%
V	EIYQAGSTPCNGAEGFNCYFPLQ	1.454	83%
E	EIYQAGSTPCNGVAGFNCYFPLQ	1.622	82%
G	EIYQAGSTPCNGVEAFNCYFPLQ	> 12.5	43%
F	EIYQAGSTPCNGVEGANCYFPLQ	> 12.5	7%
N	EIYQAGSTPCNGVEGFACYFPLQ	0.729	89%
C	EIYQAGSTPCNGVEGFNAYFPLQ	2.329	82%
Y	EIYQAGSTPCNGVEGFNCAFPLQ	3.371	63%
F	EIYQAGSTPCNGVEGFNCYAPLQ	0.295	71%
P	EIYQAGSTPCNGVEGFNCYFALQ	0.11	74%
L	EIYQAGSTPCNGVEGFNCYFPAQ	0.023	76%
Q	EIYQAGSTPCNGVEGFNCYFPLA	0.009	97%

A

	Peptide sequence	IC50 (µM)	Inhibition (%)
	EIYQAGSTPCNGVEGFNCYFPLQ	0.00136	94%
E	AIYQAGSTPCNGVEGFNCYFPLQ	0.00024	94%
I	EAYQAGSTPCNGVEGFNCYFPLQ	0.00225	94%
Y	EIAQAGSTPCNGVEGFNCYFPLQ	0.00011	92%
Q	EIYAAGSTPCNGVEGFNCYFPLQ	0.00016	94%
A	EIYQSGSTPCNGVEGFNCYFPLQ	0.00074	94%
G	EIYQAASTPCNGVEGFNCYFPLQ	0.00095	94%
S	EIYQAGATPCNGVEGFNCYFPLQ	0.00117	94%
T	EIYQAGSAPCNGVEGFNCYFPLQ	0.00022	94%
P	EIYQAGSTACNGVEGFNCYFPLQ	0.00014	94%
C	EIYQAGSTPANGVEGFNCYFPLQ	0.0056	93%
N	EIYQAGSTPCAGVEGFNCYFPLQ	0.000001	92%
G	EIYQAGSTPCNAVEGFNCYFPLQ	0.05103	92%
V	EIYQAGSTPCNGAEGFNCYFPLQ	0.00905	92%
E	EIYQAGSTPCNGVAGFNCYFPLQ	0.00346	93%
G	EIYQAGSTPCNGVEAFNCYFPLQ	0.5442	83%
F	EIYQAGSTPCNGVEGANCYFPLQ	> 12.5	18%
N	EIYQAGSTPCNGVEGFACYFPLQ	0.0229	87%
C	EIYQAGSTPCNGVEGFNAYFPLQ	0.0204	92%
Y	EIYQAGSTPCNGVEGFNCAFPLQ	0.04928	90%
F	EIYQAGSTPCNGVEGFNCYAPLQ	0.00573	92%
P	EIYQAGSTPCNGVEGFNCYFALQ	0.00053	94%
L	EIYQAGSTPCNGVEGFNCYFPAQ	0.03186	91%
Q	EIYQAGSTPCNGVEGFNCYFPLA	0.00005	94%

B

Supplementary Figure S2. The immune sera of mice immunized with $RBD_{471\text{-}493}$ were pooled (n=5) and subjected to epitope mapping using single alanine scanning competition ELISA. The ELISA was performed on plates coated with either **(A)** the native $RBD_{471\text{-}493}$ peptide or **(B)** full length Spike protein. For each inhibitory peptide, the IC50 value (µM) and the percentage of inhibition at the maximum concentration (12.5 µM) were established by comparing it with immune sera that did not contain any inhibitory peptide.

Monkey ID: CT88

	Peptide sequence	IC50 (µM)	Inhibition (%)
	EIYQAGSTPCNGVEGFNCYFPLQ	0.041	96%
E	AIYQAGSTPCNGVEGFNCYFPLQ	0.045	98%
I	EAYQAGSTPCNGVEGFNCYFPLQ	0.046	98%
Y	EIAQAGSTPCNGVEGFNCYFPLQ	0.046	97%
Q	EIYAAGSTPCNGVEGFNCYFPLQ	0.027	98%
A	EIYQSGSTPCNGVEGFNCYFPLQ		
G	EIYQAASTPCNGVEGFNCYFPLQ	0.027	98%
S	EIYQAGATPCNGVEGFNCYFPLQ	0.053	98%
T	EIYQAGSAPCNGVEGFNCYFPLQ	0.059	98%
P	EIYQAGSTACNGVEGFNCYFPLQ	0.064	97%
C	EIYQAGSTPANGVEGFNCYFPLQ	> 12.5	45%
N	EIYQAGSTPCAGVEGFNCYFPLQ	0.641	85%
G	EIYQAGSTPCNAVEGFNCYFPLQ	0.805	89%
V	EIYQAGSTPCNGAEGFNCYFPLQ	1.119	86%
E	EIYQAGSTPCNGVAGFNCYFPLQ	1.713	88%
G	EIYQAGSTPCNGVEAFNCYFPLQ	0.648	87%
F	EIYQAGSTPCNGVEGANCYFPLQ	> 12.5	35%
N	EIYQAGSTPCNGVEGFACYFPLQ	4.282	61%
C	EIYQAGSTPCNGVEGFNAYFPLQ	3.425	76%
Y	EIYQAGSTPCNGVEGFNCAFPLQ	0.012	93%
F	EIYQAGSTPCNGVEGFNCYAPLQ	0.008	89%
P	EIYQAGSTPCNGVEGFNCYFALQ	0.004	95%
L	EIYQAGSTPCNGVEGFNCYFPAQ	0.005	95%
Q	EIYQAGSTPCNGVEGFNCYFPLA	0.003	97%

Monkey ID: 112

	Peptide sequence	IC50 (µM)	Inhibition (%)
	EIYQAGSTPCNGVEGFNCYFPLQ	0.041	96%
E	AIYQAGSTPCNGVEGFNCYFPLQ	0.035	95%
I	EAYQAGSTPCNGVEGFNCYFPLQ	0.026	95%
Y	EIAQAGSTPCNGVEGFNCYFPLQ	0.035	95%
Q	EIYAAGSTPCNGVEGFNCYFPLQ	0.033	95%
A	EIYQSGSTPCNGVEGFNCYFPLQ		
G	EIYQAASTPCNGVEGFNCYFPLQ	0.024	95%
S	EIYQAGATPCNGVEGFNCYFPLQ	0.057	96%
T	EIYQAGSAPCNGVEGFNCYFPLQ	0.044	95%
P	EIYQAGSTACNGVEGFNCYFPLQ	0.053	94%
C	EIYQAGSTPANGVEGFNCYFPLQ	> 12.5	11%
N	EIYQAGSTPCAGVEGFNCYFPLQ	0.001	91%
G	EIYQAGSTPCNAVEGFNCYFPLQ	> 12.5	45%
V	EIYQAGSTPCNGAEGFNCYFPLQ	> 12.5	43%
E	EIYQAGSTPCNGVAGFNCYFPLQ	0.152	92%
G	EIYQAGSTPCNGVEAFNCYFPLQ	0.169	72%
F	EIYQAGSTPCNGVEGANCYFPLQ	> 12.5	7%
N	EIYQAGSTPCNGVEGFACYFPLQ	0.391	74%
C	EIYQAGSTPCNGVEGFNAYFPLQ	3.491	69%
Y	EIYQAGSTPCNGVEGFNCAFPLQ	0.010	90%
F	EIYQAGSTPCNGVEGFNCYAPLQ	0.001	88%
P	EIYQAGSTPCNGVEGFNCYFALQ	0.007	94%
L	EIYQAGSTPCNGVEGFNCYFPAQ	0.000	94%
Q	EIYQAGSTPCNGVEGFNCYFPLA	0.032	95%

Monkey ID: 408

	Peptide sequence	IC50 (µM)	Inhibition (%)
	EIYQAGSTPCNGVEGFNCYFPLQ	0.438	97%
E	AIYQAGSTPCNGVEGFNCYFPLQ	0.141	97%
I	EAYQAGSTPCNGVEGFNCYFPLQ	0.094	97%
Y	EIAQAGSTPCNGVEGFNCYFPLQ	0.138	97%
Q	EIYAAGSTPCNGVEGFNCYFPLQ	0.154	97%
A	EIYQSGSTPCNGVEGFNCYFPLQ		
G	EIYQAASTPCNGVEGFNCYFPLQ	0.050	97%
S	EIYQAGATPCNGVEGFNCYFPLQ	0.143	97%
T	EIYQAGSAPCNGVEGFNCYFPLQ	0.112	97%
P	EIYQAGSTACNGVEGFNCYFPLQ	0.003	97%
C	EIYQAGSTPANGVEGFNCYFPLQ	> 12.5	-7%
N	EIYQAGSTPCAGVEGFNCYFPLQ	0.003	97%
G	EIYQAGSTPCNAVEGFNCYFPLQ	> 12.5	10%
V	EIYQAGSTPCNGAEGFNCYFPLQ	> 12.5	10%
E	EIYQAGSTPCNGVAGFNCYFPLQ	3.846	83%
G	EIYQAGSTPCNGVEAFNCYFPLQ	0.002	97%
F	EIYQAGSTPCNGVEGANCYFPLQ	> 12.5	-3%
N	EIYQAGSTPCNGVEGFACYFPLQ	0.367	95%
C	EIYQAGSTPCNGVEGFNAYFPLQ	> 12.5	34%
Y	EIYQAGSTPCNGVEGFNCAFPLQ	0.075	97%
F	EIYQAGSTPCNGVEGFNCYAPLQ	0.175	96%
P	EIYQAGSTPCNGVEGFNCYFALQ	0.083	96%
L	EIYQAGSTPCNGVEGFNCYFPAQ	0.003	97%
Q	EIYQAGSTPCNGVEGFNCYFPLA	0.055	96%

Supplementary Figure S3. Epitope mapping of immune sera from vaccinated individual Macaca fascicularis was performed by single alanine scanning competition ELISA. Figure illustrates the IC50 (µM) value and the percent of inhibition of antibody binding to a non-mutant $RBD_{471-493}$ peptide with mutated peptides.
